# Intersubunit Ionic Interactions Stabilize the Nucleoside Diphosphate Kinase of *Mycobacterium tuberculosis*

**DOI:** 10.1371/journal.pone.0057867

**Published:** 2013-03-05

**Authors:** Florian Georgescauld, Lucile Moynié, Johann Habersetzer, Laura Cervoni, Iulia Mocan, Tudor Borza, Pernile Harris, Alain Dautant, Ioan Lascu

**Affiliations:** 1 IBGC, University Bordeaux, Bordeaux, France; 2 IBGC, CNRS UMR 5095, Bordeaux, France; 3 Dipartimento di Scienze Biochimiche “A. Rossi Fanelli”, Università degli Studi “La Sapienza”, Roma, Italy; 4 Laboratoire de Chimie Structurale des Macromolécules, CNRS URA 2185, Institut Pasteur, Paris, France; 5 Department of Biology, University of Copenhagen, Copenhagen, Denmark; Centro Nacional de Biotecnologia - CSIC, Spain

## Abstract

Most nucleoside diphosphate kinases (NDPKs) are hexamers. The C-terminal tail interacting with the neighboring subunits is crucial for hexamer stability. In the NDPK from *Mycobacterium tuberculosis* (*Mt*) this tail is missing. The quaternary structure of *Mt*-NDPK is essential for full enzymatic activity and for protein stability to thermal and chemical denaturation. We identified the intersubunit salt bridge Arg^80^-Asp^93^ as essential for hexamer stability, compensating for the decreased intersubunit contact area. Breaking the salt bridge by the mutation D93N dramatically decreased protein thermal stability. The mutation also decreased stability to denaturation by urea and guanidinium. The D93N mutant was still hexameric and retained full activity. When exposed to low concentrations of urea it dissociated into folded monomers followed by unfolding while dissociation and unfolding of the wild type simultaneously occur at higher urea concentrations. The dissociation step was not observed in guanidine hydrochloride, suggesting that low concentration of salt may stabilize the hexamer. Indeed, guanidinium and many other salts stabilized the hexamer with a half maximum effect of about 0.1 M, increasing protein thermostability. The crystal structure of the D93N mutant has been solved.

## Introduction

Nucleoside diphosphate kinases (NDPKs) catalyze the reversible transfer of the phosphoryl γ of nucleoside triphosphates to nucleoside diphosphates [1], [2]. The two-step reaction proceeds *via* a covalent intermediate, the enzyme being transiently phosphorylated on a conserved histidine residue, His^117^ in *Mycobacterium tuberculosis* NDPK (*Mt*-NDPK) [3]. In addition to their catalytic function, eukaryotic NDPKs are involved in complex regulatory functions, some of which unrelated to kinase activity. *Drosophila melanogaster* NDPK (*Dm*-NDPK, product of the *awd* gene) is essential for larvae development [4]. The isoform A of the human NDPK (NDPK-A or Nm23-H1) is an anti-metastatic protein [5], [6]. The isoform B of the human NDPK, also called Nm23-H2, is a transcription factor of the proto-oncogene *c-myc* [7] and possesses nuclease activity [8].

The gene coding for NDPK has been identified in *Mycobacterium tuberculosis* (*Mt*) by genome sequencing. *Mt*-NDPK has been shown to be active and to have secondary functions besides the kinase activity. It cleaves single strand DNA within the human *c-myc* promoter [9], acts as a GTPase-activating protein for Rho-GTPases [10] and damages the nuclear DNA when present in the nuclei of HeLa and COS-1 cells [11]. Importantly, it is cytotoxic for mammalian cells when secreted [12]. The toxicity mechanism is unknown, but may be related to tuberculosis pathology. In transfected human cells *Mt*-NDPK localizes to the nucleus [11], whereas human NDPKs localize both to the cytoplasm and the nucleus. The interesting biology of the *Mt*-NDPK prompted us to study its solution properties and stability to denaturation.

**Figure 1:**
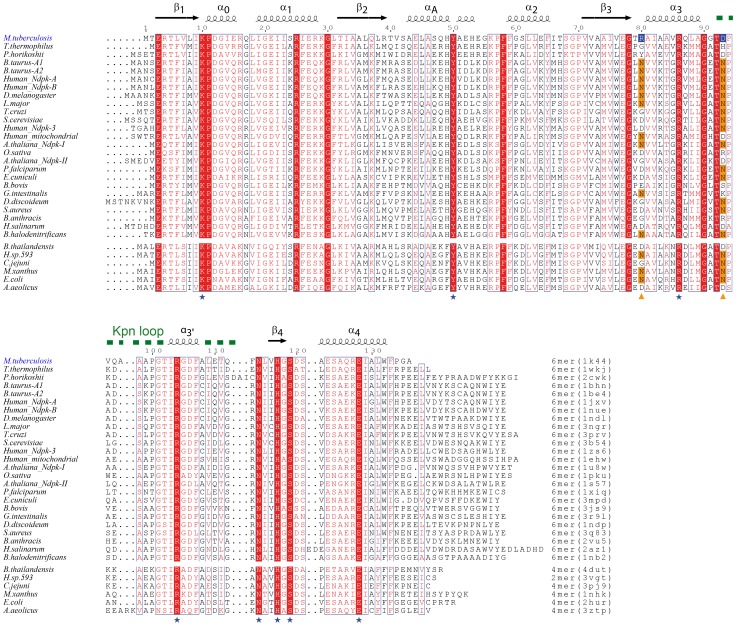
Sequence alignment of NDPKs whose structure has been solved. Sequence alignment was performed using ClustalW and mapped onto the secondary structure elements of *Mt*-NDPK, which derived from the crystal structure (PDB id 1k44) [13], by ESPript (http://espript.ibcp.fr/ESPript/ESPript/). The Kpn loop was named after the killer of prune (Kpn) mutation of Drosophila. Among the fully conserved residues indicated on red background, the activesite residues are denoted with a blue star. Triangles indicate Arg80 and Asp93 which form the salt bridge discussed in this paper. The quaternary structure and the pdb code are indicated at the end of the sequences. The enzymes of the first group from *M. tuberculosis* to *B. halodenitrificans* are hexameric, while the second tetrameric or dimeric (*H. sp*. 593).

Crystal structure of the *Mt*-NDPK has been solved [13]. It is a hexamer with a tertiary and quaternary structure very similar to other hexameric NDPKs [14]. It has been shown that several prokaryotic NDPKs are tetramers [15], [16]. Whatever the quaternary architecture, NDPKs share a common-dimer unit. Recently, such dimer unit has been found in solution for the NDPK from moderately halophilic bacteria [17], [18]. As the subunit assembly is very different in hexameric and tetrameric NDPKs, the role of the quaternary structure for protein varies between the two types of NDPKs [19]. Our study focuses on the hexameric type of NDPK enzyme. The *Mt*-NDPK protein sequence of 135 amino acids long is very similar to that of other hexameric NDPKs (>50% identity without gaps or insertions), except for a missing 15 amino acids C-terminal segment (**Figure 1**). In “long” NDPKs, this segment is extended, without secondary structure, and interacts with the neighboring subunits over about 300 Å^2^. As this interaction is repeated six-fold in the long hexamer it has a very large contribution to the overall hexamer stability. Indeed, the deletion of a few residues in the C-terminus of NDPK (cytosolic isoform) from *Dictyostelium discoideum* (*Dd*-NDPK) dramatically decreased hexamer stability [20]. The puzzling issue is that the missing interactions do not affect the *Mt*-NDPK, which is hexameric and quite thermostable, having a temperature of half denaturation (T_m_) of 73°C [13]. The molecular bases of the high stability of *Mt*-NDPK have never been understood.

One important point was to identify interaction(s) compensating the missing free energy due to the absence of the C-terminal segment. Several publications demonstrated that quaternary structure is crucial for NDPK stability and activity. We therefore focused on the analysis of intersubunit interaction. A detailed analysis failed to identify any significant differences of interfaces, between *Mt*-NDPK and other hexameric NDPKs. *Dd*-NDPK structure and properties in solution have been extensively studied. *Mt*-NDPK and *Dd*-NDPK overlap with a root mean square deviation (rmsd) rmsd of 1.0 Å distributed over the common sequence. *Dd*-NDPK having a larger subunit interface is nevertheless less thermostable than *Mt*-NDPK.

Solvent exposed salt bridges are common determinants for the thermostability of proteins [21]. The importance of specific steric and electrostatic interactions in the dimer-dimer assembly of the NDPK from moderately halophilic bacteria has been established [18], [22]. One interaction present in *Mt*-NDPK but missing in other NDPKs, is the intersubunit salt bridge Arg^80^-Asp^93^ located on the protein surface. Here, to elucidate the crucial role of that salt bridge for hexamer and overall protein stability, a mutant having the salt bridge abolished (D93N) has been prepared. The aspartate was changed to asparagine since many NDPKs have asparagine in that position. The activity, the stability and the crystal structure of the wild-type *Mt*-NDPK and the D93N mutant were compared.

## Materials and Methods

### Mutagenesis and Protein Purification

D93N gene mutation was introduced using the Transformer™ site-directed mutagenesis kit (Clonetech). The recombinant proteins *Mt*-NDPK wt and D93N mutant were expressed using a pET24 vector (Novagen) in the BL21-derived host strain BL21-CodonPlus®(DE3)-*RIL* (Stratagene). The mutation was confirmed by nucleotide sequencing and the molecular weight of proteins checked by mass spectrometry. The culture medium contained 16 g/L bacto tryptone, 10 g/L bacto yeast extract, 5 g/L sodium chloride, in the presence of 80 µg/mL of kanamicyn; expression was induced with 1 mM IPTG for 6 hours at 37°C, once the optical density reached 0.5–0.7 units. The purification steps were carried out at 4°C. After harvesting, the *E. coli* cells were sonicated and centrifuged in order to recuperate the soluble fraction containing *Mt*-NDPKs. The DNase-treated bacterial extract was applied to a Q-Sepharose column equilibrated in 100 mM Tris-HCl, pH 7.4. The enzyme was eluted at 0.5–0.6 M sodium chloride, in a linear gradient of 0–0.8 M sodium chloride in the same buffer. Active fractions were precipitated with 80% saturated ammonium sulfate and further purified by salting-out chromatography on a unmodified sepharose 6B column equilibrated with 80% ammonium sulfate, 100 mM Tris-HCl, pH 7.4. The protein was eluted by a linear gradient from 80% to 20% ammonium sulfate in the same buffer. The active fractions were pooled, dialyzed against 100 mM Tris-HCl, pH 7.4, and further purified on a Source 15Q column, under the conditions described for the Q Sepharose chromatography. The enzymes were precipitated by dialysis against a saturated solution of ammonium sulfate, recovered by centrifugation and further purified by size-exclusion chromatography on a Sephacryl S-200 column equilibrated with 0.2 M sodium phosphate buffer, pH 7.0 (Buffer A). This step allowed to cleanup the sample, by eliminating aggregated and dissociated protein.

The enzymes were essentially pure as ascertained by polyacrylamide gel electrophoresis in the presence of SDS. The concentrations of WT and mutant *Mt-*NDPKs were determined from the optical density at 280 nm using an extinction coefficient of 0.48 for 1 mg/mL, which was calculated from the amino acid composition.

### Calorimetry

Heat capacity versus temperature profiles were recorded with a VP-DSC differential scanning microcalorimeter (MicroCal Inc., Northampton, MA) at a scan rate of 1°C/min. Protein samples were diluted to 0.2–0.4 mg/mL concentration, dialyzed against buffer A and degassed before the calorimetric experiment. The reference cell was filled with degassed buffer A. Both cells were kept under an excess pressure of 30 psi to avoid bubbling during the scan. At the end of each run, the solutions were cooled and subjected to a second heating cycle under the same conditions to determine the reversibility of the transitions. Thermograms were corrected by subtracting the instrumental baseline, obtained with both cells filled with buffer A, and normalized for protein concentration. The T_m_ (temperature at which excess heat capacity reaches a maximum) and the denaturation enthalpy (ΔH) were determined with the ORIGIN software provided by MicroCal, after subtraction of a progress baseline connecting the pre- and post-transition traces. Errors are estimated to be ±0.05°C for the T_m_.

### Stability and Enzymatic Activity Measurements

For the unfolding/refolding curves, native or unfolded *Mt*-NDPK was diluted at the final protein concentration of 10 µg/mL in 0–8 M urea or 0–5 M guanidinium hydrochloride (GuHCl), and 20 mM phosphate buffer, pH 7.0 at 25°C and incubated for 16 hours. Fluorescence intensities of the single tryptophan residue Trp132 were measured at 335 nm (bandwidth of 5 nm) with an excitation at 295 nm (bandwidth of 5 nm). Data were normalized after linear fitting correction of the pre- and post-transition. Enzymatic activities were measured with the spectrophotometric assay, containing 1 mM ATP and 0.2 mM 8-bromoinosine-5′-diphosphate as substrates [23]. The errors associated with the kinetic parameters are less than 20%.

### Size-exclusion Chromatography

Size-exclusion chromatography was performed using a Superdex 75 HR 10/30 or a Superose 12 HR 10/30 column (Pharmacia, Uppsala) equilibrated with a buffer solution of 50 mM Hepes pH 7.4 containing 150 mM sodium chloride, and eluted at a flow-rate of 0.4 mL/min. The column was calibrated with a set of molecular weight markers (BioRad Markers). Protein was detected by absorbance or by fluorescence intensity at 340 nm with excitation at 280 nm (excitation and emission bandwidths of 10 nm) using a flow cell on the LS50B spectrofluorimeter (Perkin-Elmer).

### Circular Dichroism

CD ellipticity at 222 nm was recorded on a Jasco J 810 spectropolarimeter between 25 and 80°C at 1°C/min heating rate using a 1 mm quartz cuvette.

### Crystallization of the D93N Mutant

The protein solution was dialysed against 20 mM Tris-HCl, pH 7.5 buffer containing 20 mM MgCl_2_ and concentrated to 11 mg/mL. Crystallization screening was carried out using a Honeybee 961 robot (Cartesian Technology) mixing 200 nL of protein solution with 200 nL of reservoir solution (Crystal Screen, Hampton Research and The Classics Screen, Nextal). Crystals grew at 20°C in a few hours. Two different crystal forms were obtained: (i) hexagonal plates with 2.0 M ammonium sulfate, 0.1 M Tris-HCl, pH 8.5, (ii) rods with 2.0 M ammonium sulfate, 2% (v/v) PEG400, 0.1 M Hepes, pH 7.5. Crystals were cryo-protected in mother liquor supplemented with 20% glycerol (v/v) and flash-frozen in liquid nitrogen.

### X-Ray Diffraction Data Collection

Complete data sets were collected at 107 K on the ID23-2 beamline (ESRF, Grenoble), scaled with SCALA from CCP4 suite and processed with MOSFLM [24]. The structures were solved by molecular replacement with MOLREP using the coordinates of the wild type *Mt*-NDPK (PDB id: 1k44) [13] as search model. Refinement was done using phenix.refine [25] alternated with manual model building using COOT [26]. Data collection and refinement statistics are gathered in **Table 1**. The surface areas and hydrogen bonds were calculated using PISA [27]. The crystal structure was drawn using PYMOL [28].

**Table 1:** X-Ray data processing and refinement statistics.

Data collection	D93N (Form I)	D93N (Form II)
Pdb Id	4anc	4and
Space group	P4_3_32	P2_1_3
a, b, c (Å)	110.68, 110.68, 110.68	108.42, 108.42,108.42
Resolution (Å)^a^	26.90-2.80 (2.95-2.80)	26.30-2.81 (2.95-2.81)
R_sym_ a,b	0.089 (0.427)	0.081 (0.349)
R_pim_ a	0.020 (0.096)	0.027 (0.112)
I/σ(I)^a^	6.4 (1.6)	5.6 (2.1)
Completeness (%)^a^	99.9 (100.0)	99.9 (100.0)
Redundancy^a^	20.5 (21.0)	11.0 (11.1)
Solvent (%)	68.5	66.4
Matthew’s coefficient	3.93	3.69
Z	1	2
**Refinement** a		
Resolution (Å)	26.90-2.80	26.30-2.81
Highest resolution bin	3.08-2.80	3.09-2.81
Nr. reflections^a^	6 145(2 562)	10 722 (2 437)
R_work_	0.18(0.24)	0.22(0.34)
R_free_ a,c	0.22(0.29)	0.25(0.41)
**No. atoms**		
Protein	991	1958
Solvent	19	26
**B-factors**		
Wilson plot (Å^2^)	58.24	69.90
Protein (Å^2^)	68.16	74.83
Water (Å^2^)	55.11	55.32
**Rms deviations**		
Bond lengths (Å)	0.008	0.006
Bond angles (°)	1.22	1.01
Ramachandran plot*^d^*	0.0/94.0	0.0/96.1

a Statistics for the highest resolution bin are shown in parentheses. *^b^*R_sym_ were calculated by 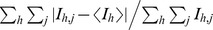, where *h* is the index for unique reflections and *j* is the index for symmetry redundant reflections. *I_h_* is the mean weighted intensity after rejection of outliers. *^c^*R_work_ and R_free_ were calculated by Σ||F_observed_|−*k*|F_calculated_||/Σ|F_observed_|. R_free_ was calculated using 5% random data omitted from refinement. *^d^*Percentage of Ramachandran outliers and favored.

### Miscellaneous

All experiments were repeated three times. The experiments were performed at 25°C in 20 mM sodium phosphate buffer (pH 7.0) unless otherwise stated. The pKa values for all acidic/basic residues based on desolvation, hydrogen bonding and charge-charge interactions were computed with PROPKA [29].

## Results

### Expression and Properties of wt and Mutant Proteins

The wt *Mt*-NDPK and D93N mutant were expressed in *E. coli.* The D93N mutant enzymes displayed catalytic properties very similar to those of the wt enzyme, within experimental errors. Mutation aspartate 93 to asparagine decreases *k*_cat_ from 264 s^-1^ in the wt enzyme to 232 s^-1^ for the D93N mutant, while the apparent *K*_m_ for 8-bromoinosine 5′-diphosphate increases from 159 µM to 230 µM (measured at a fixed concentration of 1.0 mM ATP). UV, fluorescence and CD spectra were identical for the wt and mutant enzymes (**Figures S1 and S2)**. Both proteins were hexameric as ascertained by size-exclusion chromatography (**Figure S2**). This indicates that the mutation does not affect the global structure of the *Mt*-NDPK.

### The Hexameric Structure is Necessary for Full Enzymatic Activity

The results of the fluorescence stopped flow experiments show that *Mt*-NDPK recovered the monomeric native structure within 1 second. At low protein concentration, when diluting the GuHCl-unfolded *Mt*-NDPK directly into the assay medium, the recovered specific activity of the enzyme was about 4 U/mg, which represents about 1% of the hexamer activity. Such a low activity could be attributed to monomeric or dimeric species. The hexameric structure is necessary for full enzymatic activity during its dissociation or assembly as with other NDP kinases [30], [31].

### Thermal Stability of the Wild-type *Mt*-NDPK and D93N Mutant

**Figure 2:**
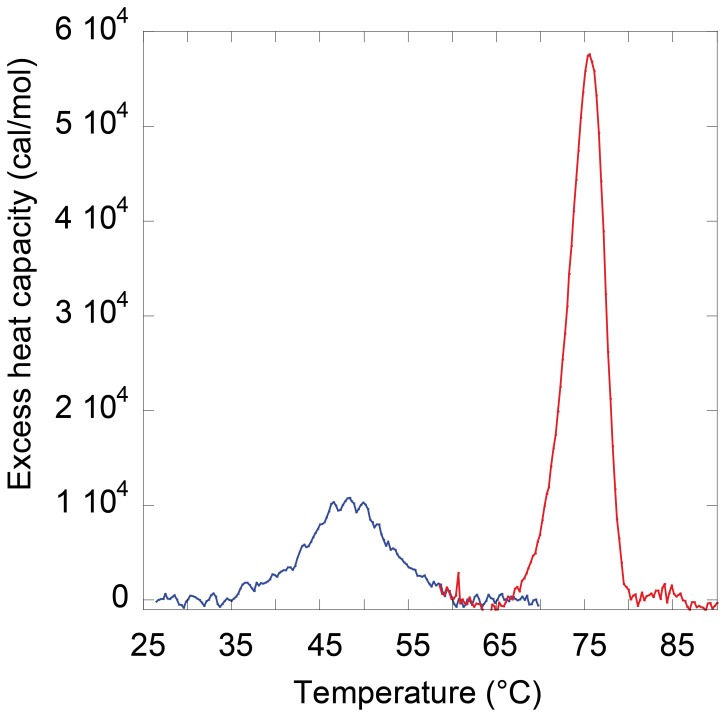
Thermostability of wild type *Mt*-NDPK and D93N mutant. The temperature dependence of excess molar heat capacity of the wild-type *Mt*-NDPK (in red) and D93N mutant (in blue). Each DSC curve displays a single calorimetric peak. The protein concentration was 0.2–0.3 mg/mL.

The differential scanning calorimetry (DSC) experiments (**Figure 2**) display only one calorimetric peak with the two proteins. The thermal stability, as measured by DSC, dramatically decreased when mutating the Asp^93^ into Asn. The T_m_ of the D93N mutant was 48.4°C while that of the wt enzyme was 76°C. No reversibility was ever observed after heat denaturation, so no complete thermodynamic analysis of the thermograms could be performed. Very close T_m_ values were obtained measuring the enzyme inactivation and the ellipticity at 222 nm (see below) under identical protein concentrations and heating rate. As the thermal denaturation was irreversible, it was less informative than the chemical denaturation.

### Stability to Chemical Denaturation

We used both urea and GuHCl as denaturants since Arg^80^ and Asp^93^ interact via an intersubunit salt bridge. The two denaturants act differently since GuHCl is a salt, while urea is a neutral molecule.

**Figure 3:**
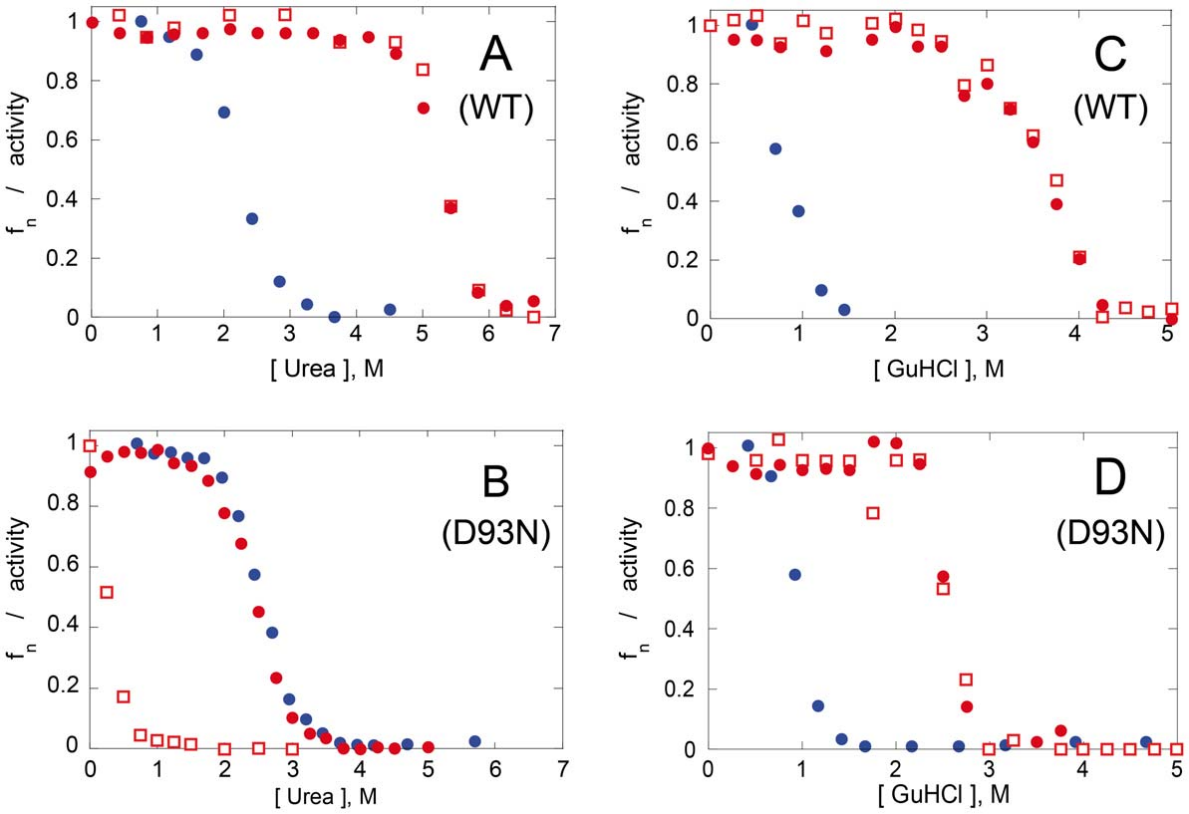
Denaturation/renaturation by urea/GuHCl. Unfolding (red circles) and subsequent refolding (blue circles) were monitored by following the intrinsic fluorescence of *Mt*-NDPK (**A** in urea, **C** in GuHCl) and D93N mutant (**B** in urea, **D** in GuHCl). The residual enzymatic activity for the unfolding was shown by red squares. The protein concentration was 10 µg/mL. The measurements are normalized to the maxima; f_n_ is the fraction of native protein.

**Figure 3A** displays denaturation transitions of wt *Mt*-NDPK in urea as measured by the fluorescence intensity change of the single tryptophan residue (indicative for the tertiary structure), as well as by enzymatic activity (indicative for the quaternary structure). Denaturation is parallel with inactivation. No dissociated species could be detected by size-exclusion chromatography. Renaturation occurred at much lower urea concentrations. Previous studies showed that hexameric NDPKs display similar hysteresis in urea after denaturation/renaturation experiments [32], [33]. The denaturation curve describes the transition from native hexamer (N_6_) to the unfolded protein (U) (Eq. 1), while the renaturation curve measured by intrinsic fluorescence intensity describes the transition from the unfolded protein to folded monomer (N) (Eq. 2).
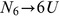
(1)

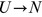
(2)

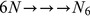
(3)


The reactivation yield was low. Hexamer formation (measured by reactivation) is the result of at least three second-order reactions (Eq. 3). This process is very slow at the low protein concentration used here [19]. For this reason the reactivation of *Mt*-NDPK was not studied. When we refolded the unfolded *Mt*-NDPK by dialysis at a much higher concentration (300 µg/mL), full reactivation was obtained with a specific activity of 550 U/mg.

**Figure 4:**
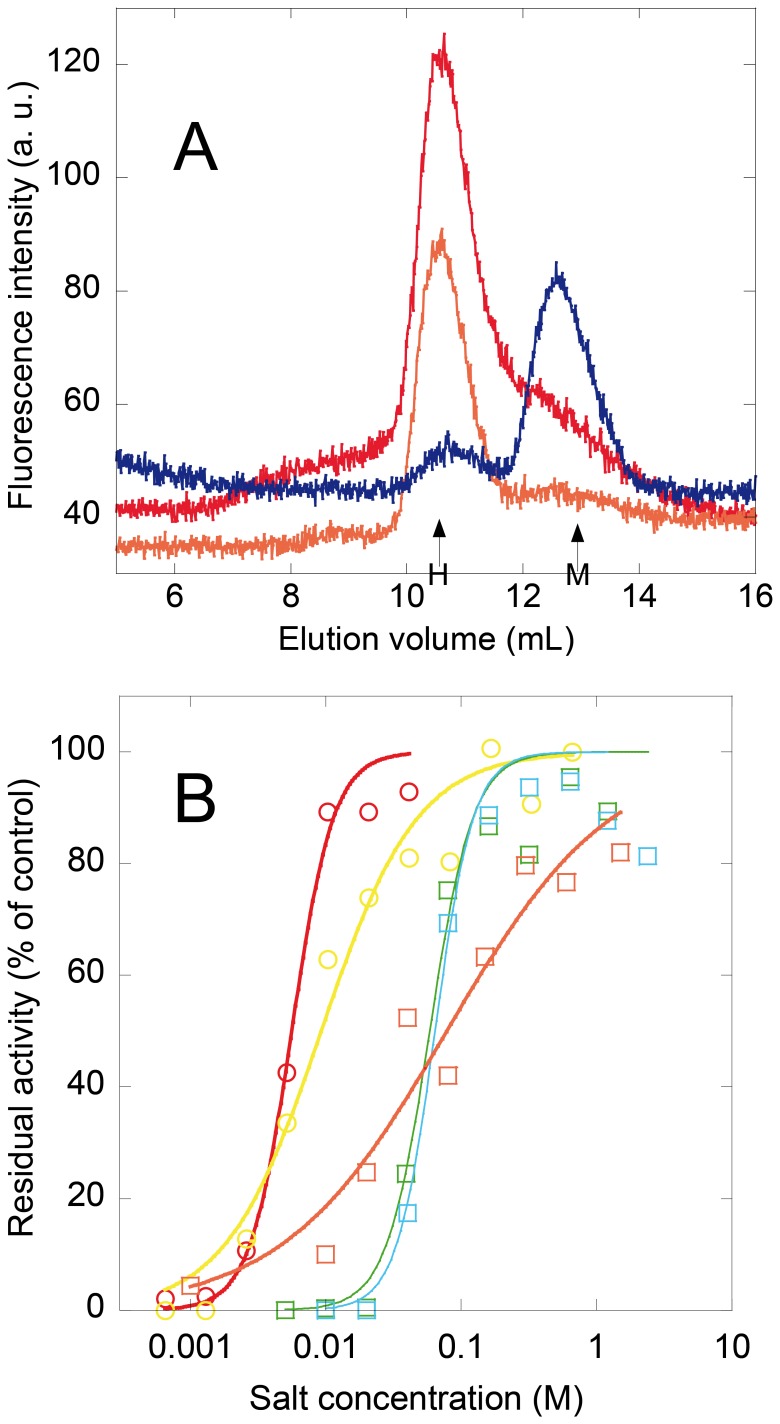
GuHCl and other salts promote association of urea-dissociated D93N mutant of *Mt-*NDPK. 100 µl of protein at 10 µg/mL was incubated for 16 h in 1.5 M urea, in the absence or the presence of salt. (**A**) Size-exclusion chromatographic analysis, with the D93N mutant in 1.5 M urea (blue), in 1.5 M GuHCl (red) and in 1.5 M urea plus 1.0 M GuHCl (orange) injected into a Superdex 75 10/300 column and the intrinsic protein fluorescence was recorded. The elution profile of *Mt*-NDPK incubated with 1.5 M GuHCl (empty circles) is shown for comparison. Expected positions for folded monomer (M, 14.5 kDa) and hexamer (H, 87.0 kDa) are indicated. (**B**) Measurement of residual activity of the D93N mutant, at 10 µg/ml was incubated for 16 h at 25°C in the presence of 1.5 M of urea plus monovalent (squares) and divalent (circles) salts: GuHCl (orange), NH_4_Cl (cyan), NaCl (green), MgCl_2_ (yellow) or CaCl_2_ (red). The enzymatic activity was measured with the standard assay. The lines do not represent theoretical models but were drawn to help the reader.

**Figure 5:**
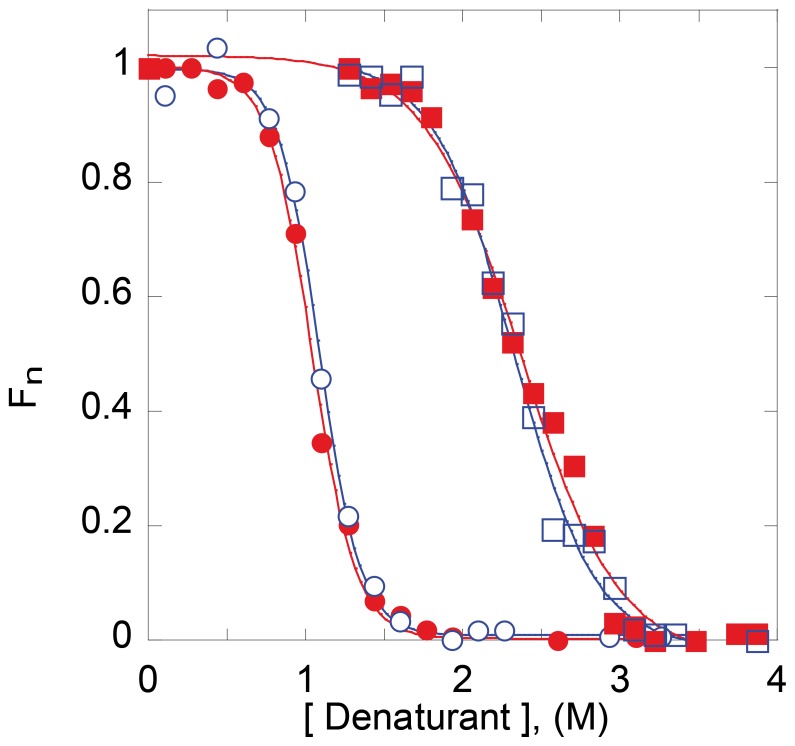
Determination of the *Mt*-NDPK stability at the monomeric state by a double dilution experiment. The *Mt*-NDPK was first unfolded in 8 M urea or 5 M GuHCl, then refolded for 10 sec by 10-fold dilution in buffer, which is sufficient to allow subunit folding but not for subunit association. Unfolding curves of *Mt*-NDPK at the monomeric state were obtained by further incubating the proteins for 16 h at 25° at the concentration of denaturant as indicated. The final protein concentration was 11 µg/mL. Circles indicate experimental data in GuHCl, while squares refer to data in urea. Red and blue symbols refer to denaturation and renaturation, respectively. f_ n_ is the fraction of native protein. The ΔGH_2_0 calculated was 4.7±0.3 kcal/mol in GuHCl and 5.0±0.5 kcal/mol in urea.

The UV and fluorescence spectra of the species that accumulated during refolding were characteristic for a native protein. Such species eluted essentially as a monomer (10% of hexamers) by size-exclusion chromatography with appropriate calibration standards (**Figure 4A**). Second, it is not a folding intermediate since it does not bind BisANS, a dye having a high affinity for the folding intermediates [33]. Moreover, an oligomeric state could also be excluded by the double dilution experiment (**Figure 5**).

In contrast, a very different pathway appeared when performing the denaturation/renaturation by urea with the D93N mutant (**Figure 3B**). Inactivation occurred at very low urea concentrations (<0.5 M). This indicates dissociation without loss of tertiary structure. This was demonstrated by size-exclusion chromatography (**Figure 4A**). Unfolding and refolding were reversible and had a midpoint of concentration for denaturation (c_1/2_) of 2.4 M urea. The denaturation curve was reversible and a ΔG_NU_ of 4.6 kcal/mol calculated. This value was very close to the ΔG_NU_ calculated for the wild-type *Mt*-MDPK by a double dilution experiment (**Figure 5**). The dramatic decrease of the protein stability was therefore due to the decrease of subunit interaction. Overall, the dissociation/denaturation of the D93N mutant can be described by Eq. 4.
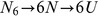
(4)


As we suspected the intersubunit salt bridge Arg^80^-Asp^93^ to be essential for the hexamer stability, we next studied the reversible dissociation/denaturation by GuHCl. In contrast with urea, GuHCl is a salt. It has been shown that ionic interactions are cancelled in GuHCl denaturation experiments while still present in the denaturation experiments by urea [34]. The wild-type *Mt*-NDPK unfolded and inactivated simultaneously in the presence of GuHCl (**Figure 3C**) as with urea as denaturant (**Figure 3A**). Refolding occured at much lower GuHCl concentrations. Again, the renatured species at 0–0.5 M GuHCl was the folded monomeric protein.

The D93N mutant displayed an unexpected behavior in the denaturation experiment with GuHCl. The hexamer was stable and active up to 2.5 M GuHCl (**Figure 3D**) and refolded had a c_1/2_ of 0.95 M GuHCl. Importantly it easily dissociated in low urea concentrations (**Figure 3B**). The loss of activity along with unfolding in higher GuHCl concentrations indicate again that dissociated species did not accumulate. The refolding experiments in presence of urea and guanidinium show identical c_1/2_ refolding for the wt and D93N monomers. This indicates that the thermodynamic stability of the monomer has not been affected by the D93N mutation. The thermodynamic stability of the isolated subunits did not change by the D93N, as in urea.

### Salts Stabilize *Mt*-NDPK

The experiments shown in **Figures 3B and 3D** with the D93N mutant indicate that urea was very efficient in dissociating the hexamer to native monomer, while GuHCl was not. This is a nontrivial observation since in general GuHCl is more efficient than urea in both dissociation and unfolding of proteins. The only explanation is the stabilization of the hexamer by GuHCl at concentrations lower than denaturing. This was found indeed to be the case. 1.5 M urea dissociated the D93N mutant to folded monomers, as indicated by the size-exclusion chromatography on a calibrated column (**Figure 4A**). The folded monomer has the smallest size among all possible species (unfolded proteins, folding intermediates and oligomeric structures) and therefore no misinterpretation of the elution profile is possible. The fluorimetric detection allows us to analyze the molecular mass distribution at very low protein concentrations, the same as used for activity measurements or steady-state fluorescence analysis. The undenatured mutant eluted as a hexamer in buffer but also in the presence of 1.5 M GuHCl. The hexamer was also the major species in the presence of combined 1.5 M urea +1.0 M GuHCl (**Figure 4A**).

We took advantage of the fact that the full enzyme activity is essentially associated with the hexamer to investigate the dissociating effect of urea in the presence of salts. By incubating the D93N mutant at 10 µg/mL with 1.5 M urea, very little activity was present after 16 h of incubation. When 1.0 M GuHCl was present in the incubation mixture in addition to 1.5 M urea, the enzymatic activity reached that of the control (**Figure 4B**) and the enzyme was hexameric (**Figure 4A**). Increasing the GuHCl concentration at more than 1.5 M, activity declined since GuHCl unfolded the protein. Other salts stimulated the hexamer formation. The anion was kept constant as chloride and the cations were monovalent (sodium, ammonium and guanidinium) or divalent (calcium and magnesium) (**Figure 4B**).

**Figure 6:**
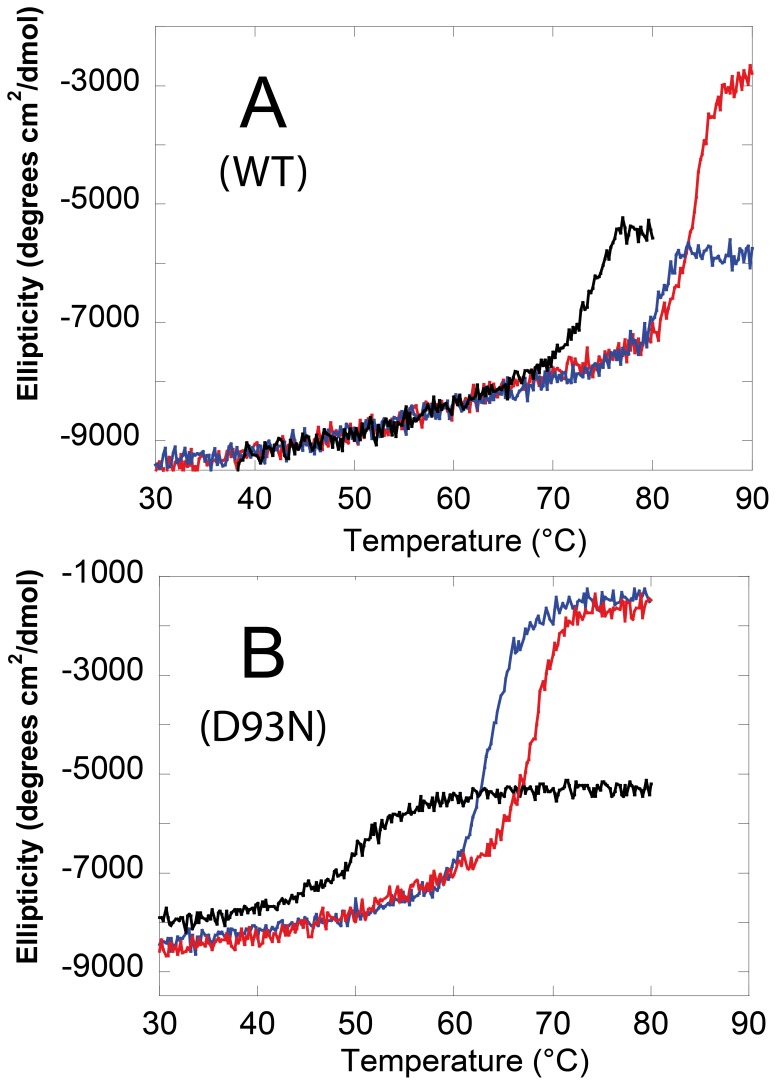
Thermal unfolding of wild-type *Mt*-NDPK and D93N mutant monitored by CD at 222 nm. The experiments were performed with wild type *Mt*-NDPK (**A**) and D93N mutant (**B**) in the absence of added salt (black) and in the presence of 0.15 M sodium chloride (blue) or 0.15 M GuHCl (red). The reduction of the absolute molar mean-residue ellipticity at 222 nm (θ_MRE_) was a measure of the loss of secondary structure.

These experiments show that many salts have an important stabilizing effect on the hexameric structure of the D93N mutant. As the quaternary structure has a major contribution to overall stability of NDPKs, we studied next the effect of salts on the thermal stability of the wild-type *Mt*-NDPK and D93N mutant (**Figure 6**).

The T_m_ of wild-type *Mt*-NDPK was 73°C in the absence of salt, 80.5°C in the presence of 0.15 M sodium chloride and 84°C in the presence of 0.15 M GuHCl (**Figure 6A**). With the D93N *Mt*-NDPK the salt effect was even more impressive. The T_m_ was 50°C in the absence of salt, 63°C with 0.15 M sodium chloride and 67°C with 0.15 M GuHCl (**Figure 6B**). It should be noted that the proteins did not unfold completely even well above the T_m_, as the ellipticity remained negative. The final CD far UV spectrum was quite similar to that of the native enzyme with reduced amplitude. Native protein was incorporated into the aggregate, or partially folded species were generated. For this reason, the quantitative thermodynamic analysis was not reliable and has not been performed.

### The D93N Mutation in *Mt*-NDPK does not Alter the 3D Structure of the Protein

**Figure 7:**
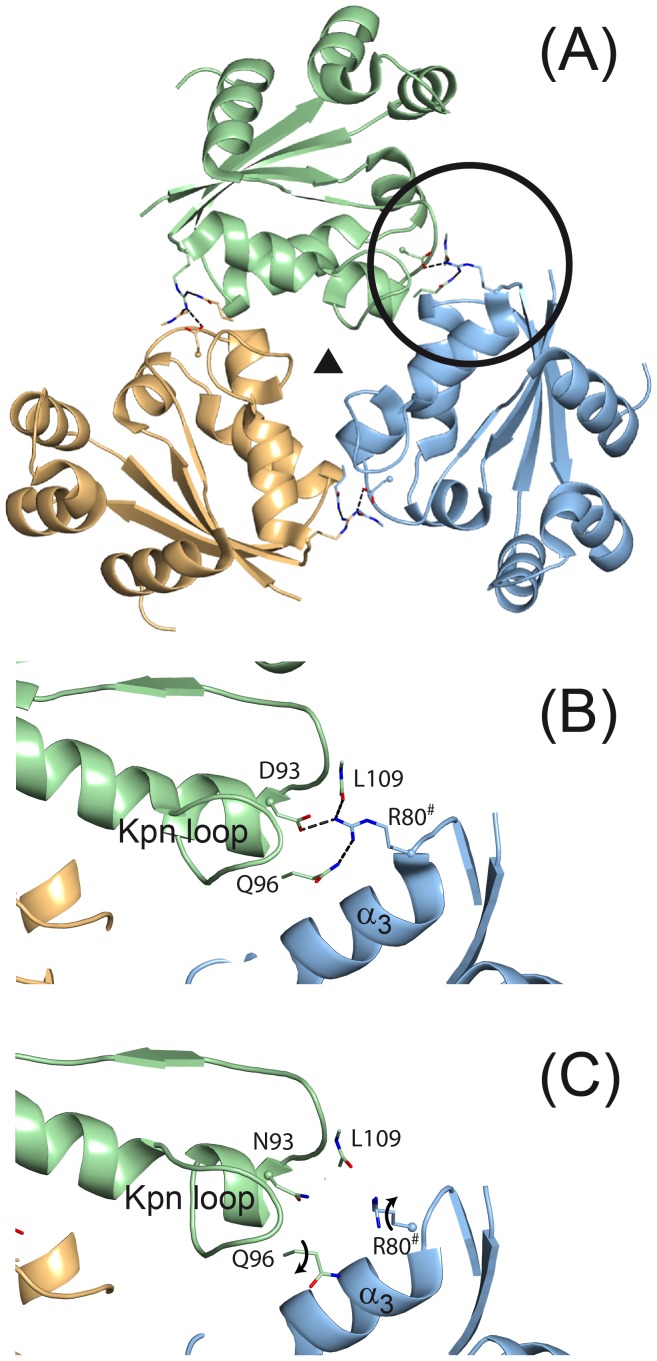
Crystal structures of wild-type *Mt*-NDPK and D93N mutant. View along the 3-fold axis of the hexamer of a “trimer” of the wild-type *Mt*-NDPK [13] (**A**). The intersubunit salt bridge found in the wild-type *Mt*-NDPK (pdb id: 1k44) (**B**) was clearly broken in the D93N mutant (pdb id: 2and) (**C**). The side-chain atoms of residues Arg^90^, Gln^96^ and Asp^93^ or Asn^93^ and the main-chain atoms of Leu^109^ were drawn as sticks. Arg^80#^ marked the arginine from the neighboring subunit. Non bonded interactions were drawn as broken lines.

In the wild-type *Mt*-NDPK hexamer, the interface between adjacent subunits was stabilized by one salt bridge and four main-chain/side-chain hydrogen bonds (**Figure 7**) [13]. Besides the intersubunit salt bridge, Arg^80^ made an intersubunit hydrogen bonds with main chain carbonyl L109 and loosely with amide Q96 (**Figure 7B**).

The D93N mutant crystallizes along two different space groups (Table 1). The two structures are similar (0.40 Å of rmsd for 135 Cα) and differ only slightly in the conformation of α_A_ and α_2_ helices due to crystal contacts. The overall B-factor values of the two D93N structures (64 Å^2^ and 71 Å^2^) are similar and higher than that of the wild type (31 Å^2^). That could be related to the stability of the oligomeric assembly. Analysis of B-factor reveals that (1) two domains, the α_A_-α_2_ region (40–70) and the C_Term_ part (120–136), are highly flexible and (2) the beginning of the Kpn-loop where the mutation is located appears more flexible in the mutant than in the WT structure (**Figure S3**). In D93N structures, the intra-subunit salt bridge is obviously broken and consequently neither Arg^80^ nor Asn^93^ were involved in intersubunit hydrogen bonds (**Figure 7C**). Accordingly, the Arg^80^ and Gln^96^ side chains protruding on the surface of the hexamer are not well defined in the electron density map and appear disordered. In the mutant structure, although the overall structures of the monomer and the hexamer are essentially unaltered, the intersubunit interactions were clearly weakened.

## Discussion

### The Quaternary Structure of *Mt*-NDPK is Essential for Enzymatic Activity and for Stability

The *Mt*-NDPK active site is located between the α_2_/α_A_ helices and the Kpn-loop (amino acids 89 to 114). Amino acids participating in nucleoside binding and catalysis are very conserved in NDPKs [14]. The Kpn-loop is also involved in the contact formed by the assembly of three dimers into the enzymatically active hexamer. It is likely that in the folded monomers since the Kpn-loop is not held in place by subunit interactions, it has some mobility. This will decrease enzymatic activity as substrate binds with less efficiency. It should be noted that the enzyme *k*_cat_ as a monomer state is 1% of that of the hexamer.

NDPKs are made of small subunits (135–180 amino acids) displaying a high sequence similarity (>45% identity) (**Figure 1**). Eukaryotic NDPKs are hexamers, while bacterial NDPKs are hexamers [13] or tetramers [15], [16], [35]. In both hexameric and tetrameric NDPKs, subunit structure is identical and two subunits associate in an identical way to generate a “dimer”. It should be noted that “dimers” refer to a partial NDPK subunit association seen in the oligomer X-ray structure (tetramers or hexamer). True dimers are easily formed by tetramer dissociation in solution and are probably the basic assembly in tetrameric NDPKs. In contrast, dimers have never been observed by dissociation or during association of hexameric NDPKs. In a similar way, “trimer” refers to the assembly of three subunits in the hexamer structure and not to a trimer in solution. The discussion below will be restricted to the stability of hexameric NDPKs only. The “dimeric” interface is highly conserved in eukaryotes and bacteria [14]. The assembly of three “dimers” generates hexamers. Due to the D3 symmetry, each subunit interacts with three neighbors [14]. This makes the hexamer assembly very cooperative i.e., it can be hardly dissociated into lower-order oligomers. Most contributions to the “trimer” interface are the Kpn*-*loop and the C-terminal residues. The C-terminal tail of 15 residues of *Dd*-NDPK and other “long” NDPKs is missing in *Mt*-NDPK. Deletion of a few C-terminal amino acids in *Dd*-NDPK has been shown to greatly decrease the hexamer stability [20], [36]. The tail is devoid of secondary structure and interacts with the neighboring subunits. The “dimer” and “trimer” buried surface areas (bsa) are much lower in “short” NDPKs (**Table 2**). The quaternary structure plays an essential role in protein stability to denaturation. This has been described for dimeric proteins [37], [38] but is more predominant for higher-order oligomers [39], [40]. As a consequence, the disruption of intersubunit interfaces requires conditions which are denaturing for the dissociated subunits. Loss of quaternary structure appears simultaneously with the loss of tertiary structure. While studying the denaturation of dimeric proteins two pathways are possible: (i) the dissociation into folded monomers followed by unfolding, or (ii) the unfolding without the accumulation of dissociated species [41]. In higher order oligomers the situation is similar. Hexameric NDPKs unfold without accumulation of dissociated species. While studying refolding/association, subunit association is very slow under our protein concentration since at least three second-order reactions generate the oligomers. An apparent hysteretic phenomenon therefore appears. This is a kinetic effect and not a true hysteresis generated by a slow conformational change of the monomer [39], [42], [43]. The absence of reversibility makes thermodynamic calculations unfeasible.

**Table 2:** Structural properties of the hexameric NDPKs discussed in the text.

Organism	PDB	Nr.	rmsd	“Dimer”	“Trimer”	Hexamer	T_m_	T_m_	T_growth_
	id	a.a.	(Å)	bsa (Å^2^)	bsa (Å^2^)	bsa (Å^2^)	(°C)	[ref]	(°C)
M. tuberculosis	1k44	136	-	576	1150	10470	76	here	37
D. discoideum	1kdn	155	0.66	707	1486	13158	66	here	20–25
D. melanogaster	1nsq	153	0.83	988	1708	16800	71	[30]	25
Human (NDPK-A)	1ucn	152	0.89	985	1602	15522	58	[5]	37
T. thermophilus Hb8	1wkj	137	0.67	710	944	9924	?	-	75

The rmsd were calculated versus the wild-type *Mt*-NDPK structure [13]. Buried surface area (bsa) are calculated by subunit. The bsa is expected to contribute about 20 cal/mol for each Å^2^ of hydrophobic contact. Nr. a. a., numbers of residues in protein.

The contact area between subunits is much smaller in *Mt*-NDPK than in other hexameric NDPKs due to the absence of the C-terminal tail (**Table 2**). For this reason, when the crystal structure of the *Mt*-NDPK was solved it was a surprise that it was a hexamer. Moreover, as complex protein thermostability is related to contact area, it was further surprising that *Mt*-NDPK is as stable, or even more stable, to heat denaturation than NDPKs having much more extensive intersubunit contacts. Careful inspection of the “trimer” interface composition failed to supply any explanation why *Mt*-NDPK is quite thermostable. Interfaces are not more hydrophobic than the corresponding interfaces in *Dm*-NDPK and *Dd*-NDPK. We suggest that hexamer stability is due to the intersubunit salt bridge Arg^80^-Asp^93^.

Preliminary experiments suggest the possibility to incorporate *Mt*-NDPK into hexamers made with human NDPK, despite the differences in sequence and the absence of an interaction domain. The observed transport of *Mt*-NDPK in the nucleus of human cells [11] could be due to a cargo effect of human NDPK subunits in a mixed hexamer.

### Role of the Intersubunit Salt Bridge Arg^80^-Asp^93^ for the *Mt*-NDPK Hexamer Stability

Salt bridges located on the protein surface have been suggested to stabilize proteins from thermophilic and hyperthermophilic organisms [21], [44]. Intersubunit salt bridges have been shown to have a major contribution to overall stability of some proteins to denaturation [45], [46]. One such in *Mt*-NDPK is the intersubunit salt bridge Arg^80^-Asp^93^ [13]. This interaction is missing in most NDPKs (**Figure 1**) but is present in all NDPKs from *Mycobacteria.* Ionic interactions may contribute to a large extent to protein stability since they are efficient at a much longer distance than van der Waals interactions. For these reasons we decided to study the contribution of the Arg^80^-Asp^93^ salt bridge to the stability of *Mt*-NDPK, by mutating Asp^93^ into neutral asparagine.

Replacement of the Asp^93^ with the neutral asparagine showed a dramatically decrease of the thermal stability. The T_m_ measured by DSC drops from 76°C to 48°C. Here again, the hexamer integrity has been followed measuring the residual activity, while DSC and CD signals were due to unfolding. The three techniques supplied similar T_m_ for the wild-type and mutant *Mt*-NDPK indicating simultaneous dissociation/unfolding.

The chemical denaturation studies with urea and GuHCl as denaturants showed a large decrease in hexamer stability as a result of the D93N mutation, while the stability of the isolated subunit was not affected. This is not surprising since Asp^93^ is located on the subunit surface.

The most significant information on the effect of the mutations on hexamer stability was obtained when studying *Mt*-NDPK denaturation by urea. The wild-type inactivated/unfolded c_1/2_ was about 5.2 M. The inactivation (loss of quaternary structure) and the intrinsic tryptophan fluorescence intensity change (loss of tertiary structure) were concomitant, which suggests that the isolated subunits are not stable under the conditions needed for dissociation, or the hexamer unfolds without dissociation. The two patterns cannot be distinguished under our experimental conditions. Comparing the stability of the hexamer with that of isolated subunits reveals the important role of the quaternary structure to stabilize the overall protein native structure. We showed previously that in acidic conditions, isolated monomers of *Dd*-NDPK are unstable and form molten globule folding intermediates, while the hexamer conformation stays unchanged [47]. Moreover, isolated subunits of human NDPK-A cannot be native [48], while the native hexamer is quite stable. During *Mt*-NDPK renaturation, only the folded monomer was detected but no higher-order dissociated species such as dimers or trimers. One may speculate that evolution pressure acts on the hexamer stability and not on a single “partial” interaction. The D3 hexamer is very cooperative since each subunit has contacts with 3 other subunits. The hexamer is very stable even if all individual subunit-subunit interactions are rather weak.

In presence of urea, the mutation of the negatively charged Asp^93^ into the neutral Asn had a dramatic effect on the hexamer stability. : the c_1/2_ of the wt hexamer decreased from 5.2 M to less than 0.5 M for the D93N mutant. Folded monomers presented a c_1/2_ of 2.5 M urea for the wt as well as for the mutant. The hexamer stability decrease was therefore not due to subunit destabilization (see also **Figure 6**). In the crystal structure of D93N mutant, no direct interaction of the Asn 93 exists with neighbouring subunit.

Long-range ionic interactions may also be involved with charged residues. Based on these interactions, PROPKA software calculates a rough estimate for the free energy of unfolding. When changing an amino acid residue, the interactions changes and so does the free energy of unfolding. The calculated ΔG for the *Mt*-NDPK hexamer was 142.9 kcal/mol. It decreased to 124.4 kcal/mol for the mutant D93N, respectively. It appears that the stability calculated from PROPKA software actually corresponds qualitatively with the measured T_m_ or with the hexamer stability in urea as denaturant. The D93N mutation appears to decrease the protein stability because the negative charge of the Asp^93^ interacts with distant protein charges and stabilizes the hexamer.

### Role of Cation Binding for Hexamer Stability

The larger stability of the D93N hexamer in GuHCl than in urea is very surprising. Unexpectedly GuHCl stabilizes the hexamer, at low concentrations. Other monovalent (Na^+^ and NH_4_^+^) or divalent (Mg^+^ and Ca^+^) cations also stabilize the hexamer. The guanidinium cation is effective at slightly lower concentrations than Na^+^ or NH_4_^+^, while keeping the anion constant. GuHCl is denaturing at higher concentrations, while it is stabilizing up to 1 M. This feature explains why GuHCl is much less efficient in dissociating the D93N mutant *Mt*-NDPK. Guanidinium cation is large and hydrated and should therefore interact better with the protein surface than small cations.

Protein stabilization by monovalent cations is not frequent but some examples have been described [49], [50]. The stabilization mechanism of *Mt*-NDPK is unknown, but we suppose to be related to cation binding to the protein rather than an effect mediated by the change of global solvent properties. Indeed, cations which are on the opposite ends of the Hofmeister series are stabilizing, with similar efficiencies. Second, the effect is half-maximum effect at about 100 mM. It is however too high for measuring binding affinity and stoichiometry. This is much lower than the common stabilization mediated by a global solvent effect, which appears at molar salt concentrations. The hexamer formation with the D93N mutant in 1.5 M urea was an original way to detect and quantify the stabilizing effect in a functional way.

### Conclusions

The most important conclusion of this study was that the quaternary structure is essential for enzymatic activity and for the stability to denaturation. The lower contact surfaces between subunits, as compared to other NDPKs, are compensated by the intersubunit salt bridge Arg^80^-Asp^93^. This makes the *Mt*-NDPK quite thermostable. The thermal stability of proteins measured *in vitro* cannot be discussed as an adaptation for *in vivo* conditions, with the exception of the proteins from (hyper)thermophilic organisms. *M. tuberculosis* is a mammalian parasite and therefore lives at about 37°C. Instead, the thermal stability has been correlated with the kinetic stability of proteins *in vivo* [51], [52]. A large number of proteins of *M. tuberculosis* have been shown to be relatively thermostable [53], [54], [55], [56]. *Mt*-NDPK is not an exception in this context.

## Supporting Information

Figure S1 **UV and fluorescence spectra of wild-type** ***Mt*** **-NDPK (blue) and D93N mutant (red). (A)** The UV spectra were recorded with a protein concentration of 1.19 mg/mL (WT) or 1.00 mg/mL (D93N) in 20 mM phosphate buffer, pH 7. Note that the shoulder at 300 nm is a specificity of native form of *Mt*-NDPK. **(B)** Tryptophan fluorescence (295 nm excitation) was measured at 25°C from 310 to 390 nm. The fluorescence spectra were recorded with a protein concentration of 10 µg/mL.
(TIF)

Figure S2 **CD spectrum and size-exclusion chromatography profiles of wild-type** ***Mt*** **-NDPK (blue) and D93N mutant (red). (A)** CD ellipticity spectra were recorded between 200 and 250 nm on a Jasco J810 spectropolarimeter using a 1 mm quartz cuvette. **(B)** Size-exclusion chromatography was performed using a Superose 12 column (Pharmacia, Uppsala) equilibrated with a buffer solution of 50 mM Hepes, pH 7.4 containing 150 mM sodium chloride, and eluted at a flow-rate of 0.4 mL/min. The column was calibrated with a set of molecular weight markers: immunoglobulin (1) ovalbumin (2) and myoglobin (3) (BioRad Markers).
(TIF)

Figure S3 **Normalized Bfactor plots (B - <B>/σ(B)) of the α-carbon atoms of the six chains of the wild-type** ***Mt*** **-NDPK structure (blue) and of the three chains of D93N mutant structures (red).** Analysis of B-factor reveals that (1) two domains, the α_A_-α_2_ region (40–70) and the C_Term_ part (120–136), are highly flexible and (2) the beginning of the Kpn-loop where the mutation is located appears more flexible in the mutant than in the WT structure. The σ(B) of the two D93N structures (Pdb_Id: 4anc, 4and) and of the WT structure (Pdb_Id: 1k44) are 29 Å^2^, 25 Å^2^ and 13 Å^2^, respectively.
(TIF)
