# Supplementary figures and images for: Intersubunit Ionic Interactions Stabilize the Nucleoside Diphosphate Kinase of Mycobacterium tuberculosis
Source: PLoS One. 2013 Mar 5;8(3):e57867. doi: 10.1371/journal.pone.0057867 (PMC3589492; doi:10.1371/journal.pone.0057867)

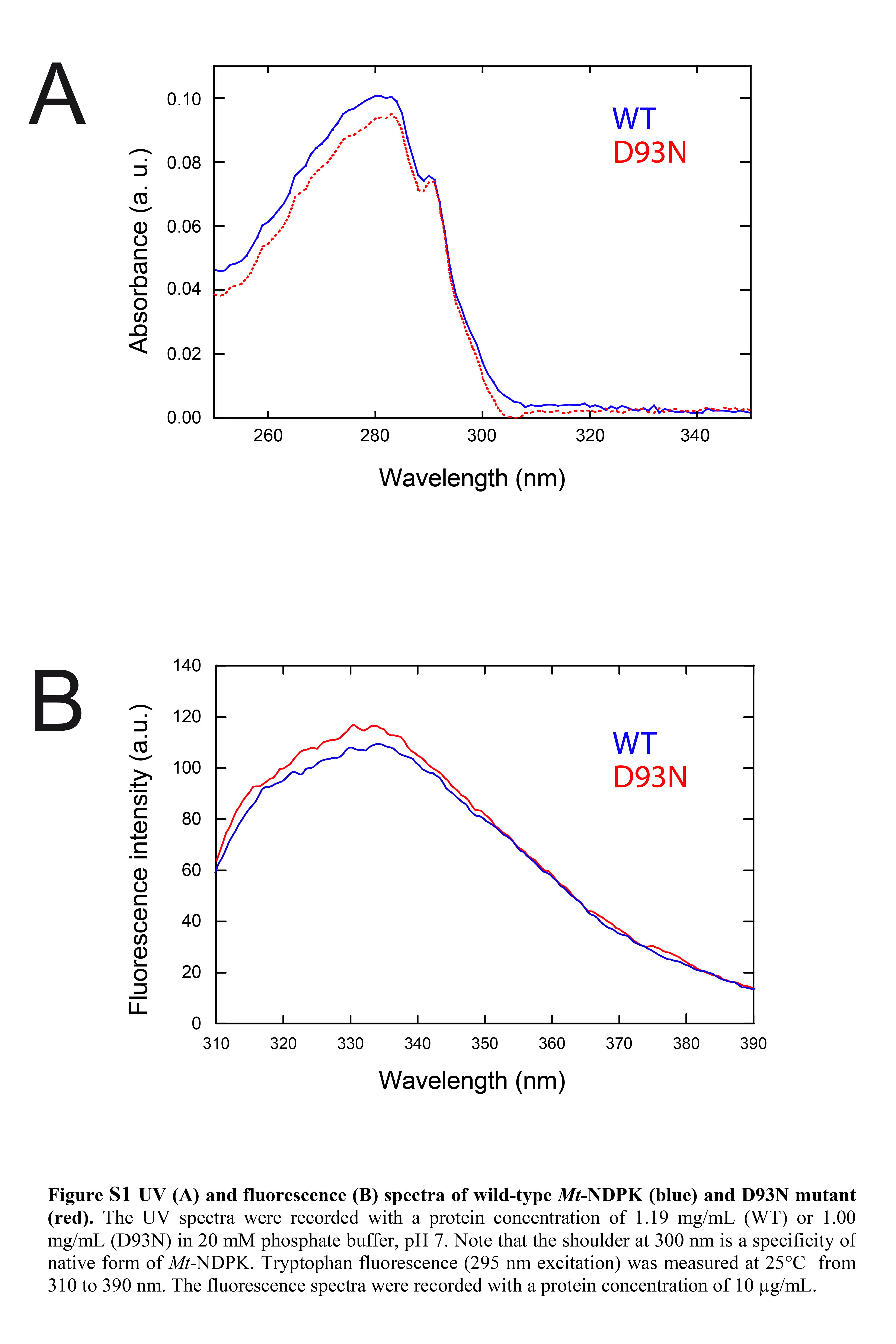

Supplement: Figure S1 — UV and fluorescence spectra of wild-type Mt -NDPK (blue) and D93N mutant (red). (A) The UV spectra were recorded with a protein concentration of 1.19 mg/mL (WT) or 1.00 mg/mL (D93N) in 20 mM phosphate buffer, pH 7. Note that the shoulder at 300 nm is a specificity of native form of Mt-NDPK. (B) Tryptophan fluorescence (295 nm excitation) was measured at 25°C from 310 to 390 nm. The fluorescence spectra were recorded with a protein concentration of 10 µg/mL. (TIF) [file pone.0057867.s001.tif]

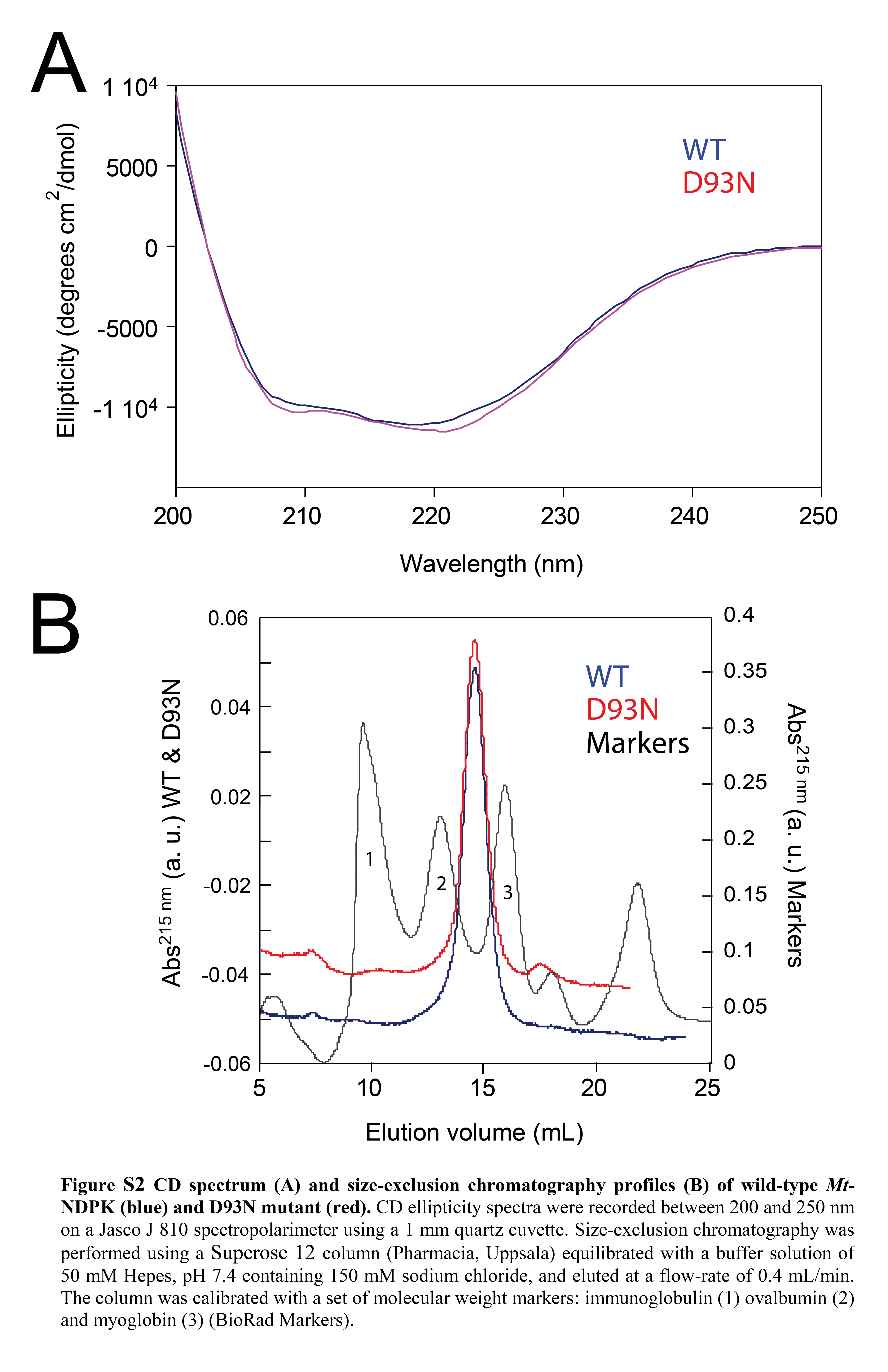

Supplement: Figure S2 — CD spectrum and size-exclusion chromatography profiles of wild-type Mt -NDPK (blue) and D93N mutant (red). (A) CD ellipticity spectra were recorded between 200 and 250 nm on a Jasco J810 spectropolarimeter using a 1 mm quartz cuvette. (B) Size-exclusion chromatography was performed using a Superose 12 column (Pharmacia, Uppsala) equilibrated with a buffer solution of 50 mM Hepes, pH 7.4 containing 150 mM sodium chloride, and eluted at a flow-rate of 0.4 mL/min. The column was calibrated with a set of molecular weight markers: immunoglobulin (1) ovalbumin (2) and myoglobin (3) (BioRad Markers). (TIF) [file pone.0057867.s002.tif]

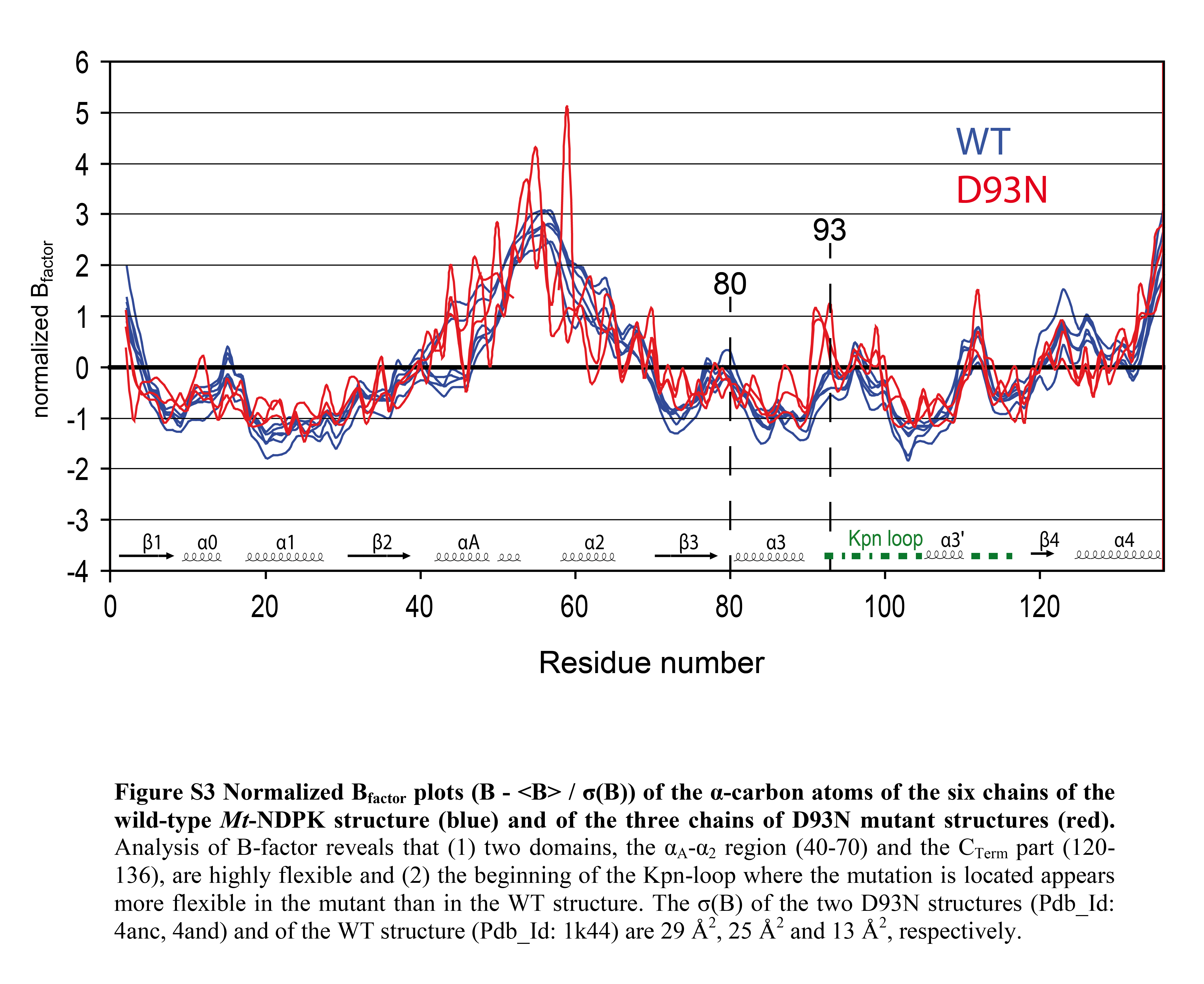

Supplement: Figure S3 — Normalized Bfactor plots (B - /σ(B)) of the α-carbon atoms of the six chains of the wild-type Mt -NDPK structure (blue) and of the three chains of D93N mutant structures (red). Analysis of B-factor reveals that (1) two domains, the αA-α2 region (40–70) and the CTerm part (120–136), are highly flexible and (2) the beginning of the Kpn-loop where the mutation is located appears more flexible in the mutant than in the WT structure. The σ(B) of the two D93N structures (Pdb_Id: 4anc, 4and) and of the WT structure (Pdb_Id: 1k44) are 29 Å2, 25 Å2 and 13 Å2, respectively. (TIF) [file pone.0057867.s003.tif]
